# Glucose oxidase converted into a general sugar-oxidase

**DOI:** 10.1038/s41598-022-14957-6

**Published:** 2022-06-23

**Authors:** Yael Baruch-Shpigler, David Avnir

**Affiliations:** Institute of Chemistry and the Center for Nanoscience and Nanotechnology, The Hebrew University of Jerusalem, 9190401 Jerusalem, Israel

**Keywords:** Enzymes, Metals

## Abstract

Entrapment of glucose oxidase (GOx) within metallic gold converts this widely used enzyme into a general saccharide oxidase. The following sugar molecules were oxidized by the entrapped enzyme (in addition to d-glucose): fructose, xylose, l-glucose, glucose-6-phosphate, sucrose, lactose, methylglucoside, and the tri-saccharide raffinose. With the exception of raffinose, none of these sugars have a natural specific oxidase. The origin of this generalization of activity is attributed to the strong protein-gold 3D interactions and to the strong interactions of the co-entrapped CTAB with both the gold, and the protein. It is proposed that these interactions induce conformational changes in the channel leading to the active site, which is located at the interface between the two units of the dimeric GOx protein. The observations are compatible with affecting the specific conformation change of pulling apart and opening this gate-keeper, rendering the active site accessible to a variety of substrates. The entrapment methodology was also found to increase the thermal stability of GOx up to 100 °C and to allow its convenient reuse, two features of practical importance.

## Introduction

Glucose oxidase (GOx) is one of the most studied and utilized enzymes—in the past two decades it appeared as a topic in more than 12,000 publications (ISI database). The enzyme has been applied in a variety of fields, including glucose sensors, biofuel production, fuel cells, food industry, textile industry, dentistry, and more^1–3^. The origins of this wide scope of applications have been GOx’s relatively low cost, its stability, the ease of assaying its activity, and the abundance and central role of d-glucose. No wonder it was termed “an ideal enzyme”^4^. Perhaps the only limiting property of GOx is its very high saccharide specificity—it operates on d-glucose only^5,6^. “Limiting”, because while for operating as a component of the very complex network of enzymes and biomolecules of life systems, specificity is an evolutionary optimization^7^, chemistry, in general, looks also for wide-scope biocatalysts as “on-the shelf” tools which can be used for a myriad of diverse needs^8,9^. Widening the ability of enzymes to operate on more than the prime natural substrate, has been termed “promiscuous”^10^, but that negative-connotation adjective overshadows the usefulness of having a wide scope enzyme, hand in hand with its high-specificity version. For instance, such widening of activity may be beneficial for a multi-substrate enzymatic biofuel cell as suggested in this report.

**Figure 1:**
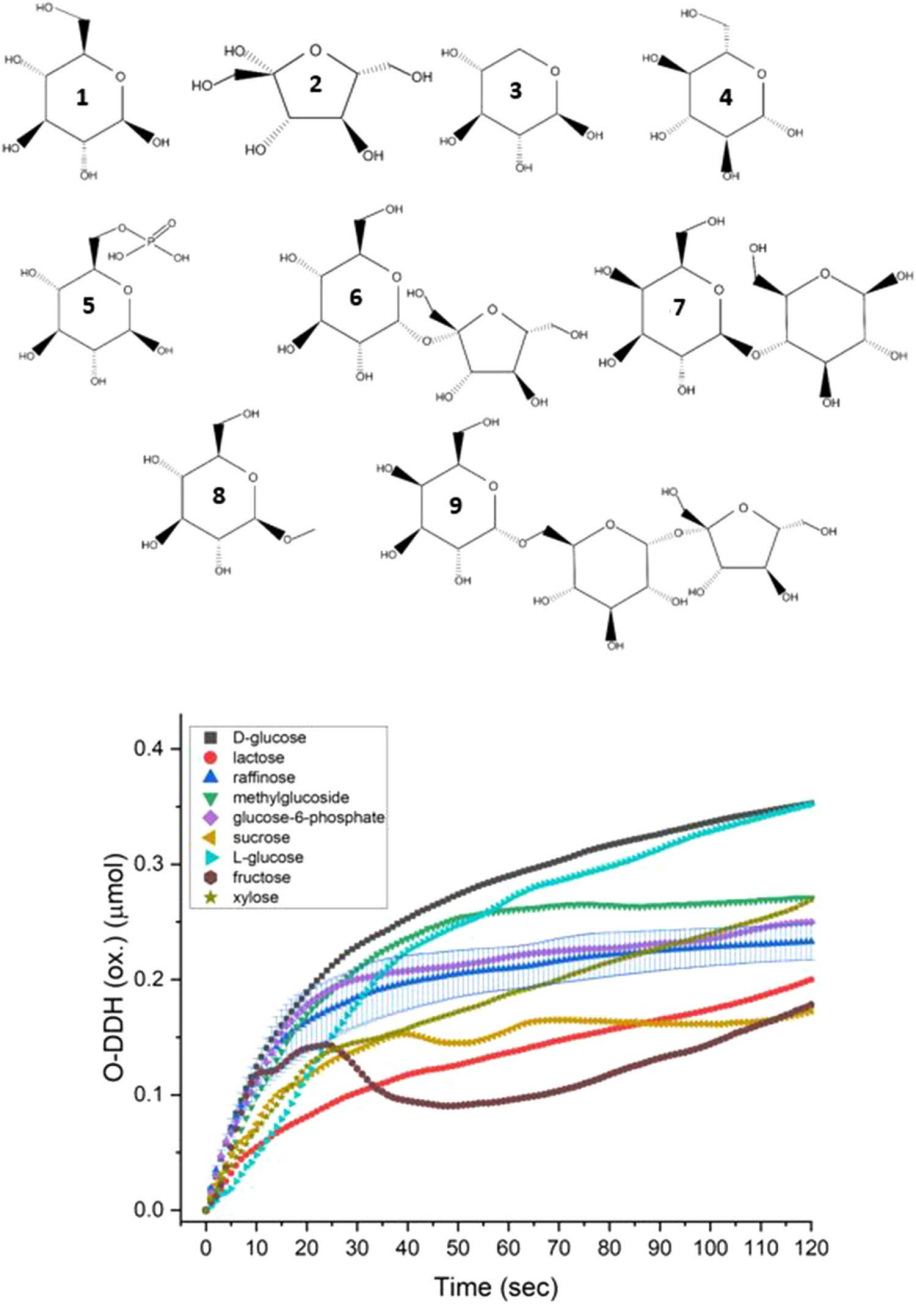
Top: structures of all of the saccharides used in the study—(1) d-glucose, (2) fructose, (3) xylose, (4) l-glucose, (5) glucose-6-phosphate, (6) sucrose, (7) lactose, (8) methylglucoside, (9) raffinose. Bottom: the activity of GOx/CTAB@Au with all of the saccharides, as a measure of oxidation of o-dianisidine (O-DDH). (Typical error bars are shown for raffinose. Error bars for methyl glucoside are shown in see Fig. 7 and for all other sugars in the Supplementary Material Figs. S1–S7).

In an earlier report^11^, we have shown that the entrapment of GOx within a 3D metallic matrix of gold, enables the extension of its activity to fructose, xylose and l-glucose, apparently due to conformational changes that open the channel leading to the two active sites of this dimeric protein. That preliminary observation pointed out to the possibility that the active site of GOx, if the right conditions are found to expose it by removing penetration restrictions, has the potential to operate on *any* desired sugar molecule, including saccharides that lack a natural specific oxidase. Here we report that the conditions for that generalization of activity have been found. Specifically we report that the co-entrapment of GOx with the surfactant CTAB (cetyltrimethylammonium bromide) within gold—GOx/CTAB@Au—expands dramatically the oxidase activity of that enzyme, not only further enhancing the activity towards the above mentioned monosaccharides, but also opening the activity towards the disaccharide lactose, the trisaccharide raffinose, the substituted saccharide d-glucose-6-phosphate, and saccharides where the d-glucose keto-enol tautomerism of the β C(1)H-OH group is blocked, namely d-methylglucoside and the disaccharide sucrose (Fig. 1). It is important to note that except for raffinose^12^, none of the sugar molecules successfully utilized here has a naturally occurring specific oxidase, that is none has an enzyme which oxidizes it directly with atmospheric oxygen^12^.

The methodology of the entrapment of enzymes in gold is based on the reduction a gold salt in the presence of the enzyme to be entrapped^13^. The result is a metallic gold matrix within which the enzyme molecules are entrapped in 3D cages, formed by the gold aggregated nanocrystallites. The enzyme molecules are tightly held and cannot leach out, and yet are accessible to reaction through the interstitial pores network of the aggregated gold. Enzymes which were successfully entrapped in gold by that method in addition to GOx, include l-asparaginase, collagenase, and horseradish peroxidase (HRP)^13^. Significantly enhanced stability of the enzymes is one of the outcomes of that type of entrapment, which is completely different from the common 2D anchoring enzymes to metal surfaces, where the enzyme molecules remain exposed to the environment^14^. This observation follows the well documented enhancement of stability of enzymes by their entrapment in 3D silicate sol–gel matrices^14–17^, a stability which is even more enhanced by the co-entrapment with CTAB^18^. And indeed, that co-entrapment proved to be beneficial also for the entrapment in gold: laccase could be kept active only when subjected to this co-entrapment^13^. The expectation that solving the laccase problem by that approach might also open the way to convert GOx into a much more general sugar oxidase was perhaps a long shot, but it was based on the special features of CTAB-protein and CTAB-gold interactions, explained in detail in the “Discussion” section.

Some comments on the sugar molecules used in this study are in order: glucose-6-phosphate (Fig. 1(5)), is naturally occurring in mammalian cells^19^ and is utilized by the body as a carbon source in a variety of processes. Methylglucoside^20,21^ (Fig. 1(8)) is employed as an intermediate in the production of plasticizers, surfactants and more^22–24^. Lactose (Fig. 1(7)), a di-saccharide composed of glucose and galactose units, is mostly used in the food industry^25^. Raffinose^26^ (Fig. 1 (9)), a tri-saccharide composed of galactose, glucose and fructose units, is found in grains and vegetables^27^, but not the human body*.* The enantiomeric l-glucose is not naturally occurring and was used in this study to gain insight as to the mechanism which widens the scope of activity of the entrapped GOx. Fructose, xylose, lactose and sucrose are well-known high-volume sugars which need no further introduction.

The wide scope of oxidase activity reported here for GOx/CTAB@Au is, to the best of our knowledge, the most intensive broadening of activity of a highly selective enzyme reported so far, without changing its primary structure. In the “Discussion” we propose that the main conformational change that the entrapped enzyme undergoes, and which is compatible with the wide scope of activity, is the widening of the channel leading to the active site. That is, that the high stereoselectivity of GOx towards d-glucose is mainly due to the stereo- and enantioselective properties of that gate, and that the active site, once reached, is an efficient oxidizer of CH-OH in a variety of saccharide structures.

## Results

### The generality of GOx as a saccharide oxidase

The generality of GOx as an oxidase of saccharides when entrapped in gold with CTAB, is shown in Fig. 1 (bottom): eight different sugar molecules (and d-glucose), representing the variety of this key family of biomolecules, are air-oxidized, none of which (except raffinose) as mentioned above, has a naturally occurring specific oxidase. Seen in Fig. 1 are mono-, di- and tri-saccharides, as well as sugar derivatives including the charged, sterically hindered phosphate derivative. None of these saccharides (except d-glucose of course) is oxidized by GOx in solution (except some low activity towards methylglucoside described next).

**Figure 2:**
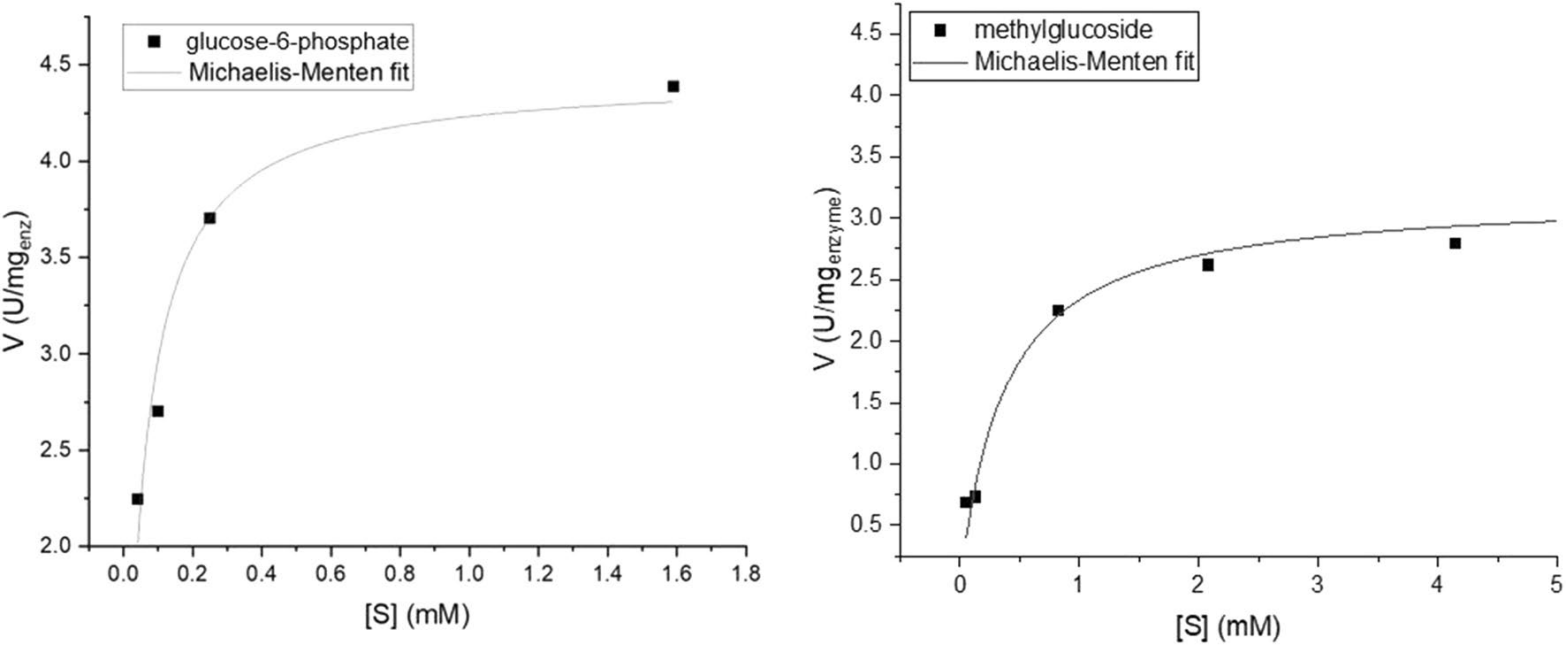
Michaelis–Menten (MM) graphs of glucose-6-phosphate (left) and methylglucoside (right).

All activities obey the Michaelis–Menten (MM) kinetic model. Apparent K_M_ and V_max_ values are collected in Table 1, and representative MM graphs are shown in Fig. 2. As seen in the Table, the entrapped enzyme is active towards all substrates, and the kinetic parameters which characterize these oxidations, do not vary wildly—less than an order of magnitude: in the cases of the monosaccharaides and of lactose (a di-saccharide) K_M_ and V_max_ are actually quite similar to d-glucose itself. Note also that the “wrong” enantiomer, l-glucose, has values of K_M_ = 0.02 mM and V_max_ = 2.1 U/mg compared with K_M_ = 0.01 mM and V_max_ = 2.8 U/mg for the native d-glucose, that is, the enantioselectivity of the enzyme is not absolute anymore. Larger differences are seen for the charged glucose-6-phosphate, for the bulky tri-saccharide raffinose, and for the two saccharides—methyl glucoside and sucrose—where the analogous C(1)H-OH site of d-glucose is blocked—the apparent K_M_ values are indeed higher, but the activity is quite clear, with even higher V_max_ values. As explained in the “Discussion”, an increase in both the K_M_ (lower affinity) and V_max_ (higher rate) values is compatible the efficient, non-selective catalytic step.

**Table 1:** Michaelis–Menten parameters of the activity of GOx/CTAB@Au on various saccharides.

Substrate	d-Glucose	l-Glucose	Fructose	Xylose	Lactose	Methyl-glucoside	Raffinose	Sucrose	Glucose-6-phosphate
Apparent K_M_ (mM)	0.01	0.02	0.01	0.01	0.01	0.30	0.08	0.07	0.05
V_max_ (U/mg)	2.8	2.1	1.7	2.1	1.2	3.0	5.9	5.1	4.4

### The role of CTAB

Insight into the origins of the combined effects of the gold cage and of the surfactant is provided by a comparative study of the effects of entrapment with and without the CTAB, GOx/CTAB@Au and GOx@Au. In all of the studied parameters—activity, thermal stability and recyclability (described next)—there is clear observation that the surfactant interactions are beneficial, enhancing and broadening the effects observed by the entrapment within the pure gold cage. As seen in Table 2, the activity and substrate affinity of the enzyme entrapped in gold only towards the monosaccharides is significantly enhanced by the co-entrapment with CTAB in all cases, even for d-glucose itself. MM analysis for the other sugars in Table 1, was not possible because the activity of GOx@Au towards these molecules was either zero or too low for reliable MM analysis.

**Table 2:** The increase of activity of GOx towards monosaccharides by co-entrapment of CTAB. [In brackets are the K_M_ and V_max_ values for the free enzyme in solution^13^].

Substrate	GOx@Au →GOx/CTAB@Au	
	Apparent K_M_ (mM)	V_max_ (U/mg)
d-glucose	0.5 → 0.01 [21.7]	1.8 → 2.8 [24.2]
l-glucose	5.5 → 0.02	1.2 → 2.1
Fructose	7.3 → 0.01	1.4 → 1.7
Xylose	0.9 → 0.01	0.7 → 2.1

Decrease of the K_M_ value of free enzymes upon immobilization—see first row in Table 2—is a well-documented phenomenon^28–30^. To understand it we recall that K_M_ is defined as the concentration at half the maximum velocity (V_max_/2)^31^, and so a decrease in the maximum velocity due to diffusional limitation induced by entrapment leads also to a drop in V_max_/2. This interpretation does not imply that the affinity of the active site to the substrate, as reflected by the K_M_ value, has increased.

**Figure 3:**
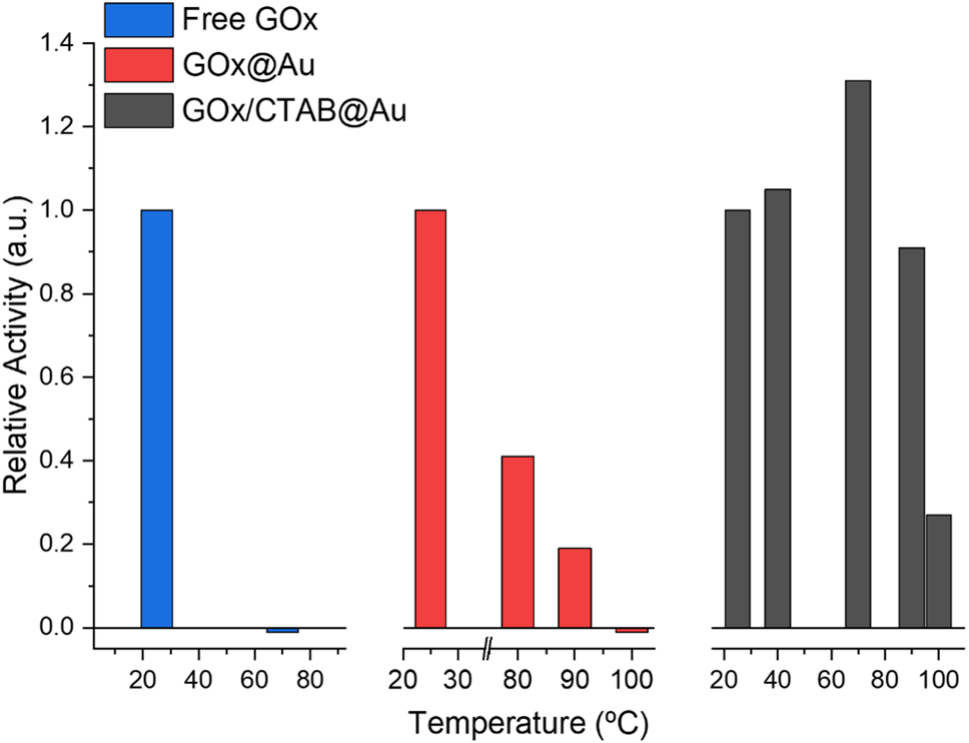
The jump in thermal stability of GOx upon its co-entrapment with CTAB within gold. Left: GOx in solution. Middle: GOx@Au. Right: GOx/CTAB@Au.

This beneficial effect is seen also for two important enzymatic activity parameters—a jump in the thermal stability and the ability to recycle. The quite dramatic increase in the thermal stability is shown in Fig. 3: in solution at 70 °C, the activity towards d-glucose drops to zero, indicating full denaturing of the enzyme. When entrapped in gold (without CTAB) the enzyme is still active at 80 °C retaining 40% of the room-temperature activity, and totally denatured at 100 °C. This feature that is much enhanced by the co-entrapment with CTAB: the thermal stability of GOx/CTAB@Au is particularly interesting—not only is there measurable activity at 100 °C, but there is an increase in activity with temperature at the range of 20–70 °C. This increase indicates that up to 70 °C the thermal driven increase in activity with temperature, characteristic of most chemical reactions, overshadows the temperature driven denaturing. Increase in thermal stability of enzymes has been observed in other 3D entrapments, particularly in sol–gel oxide matrices^15,16^, and has been attributed to the tight holding of the entrapped enzyme, diminishing the conformational re-folding which leads to the denaturing. In the discussion, we detail the interactions that lead to this stability.

**Figure 4:**
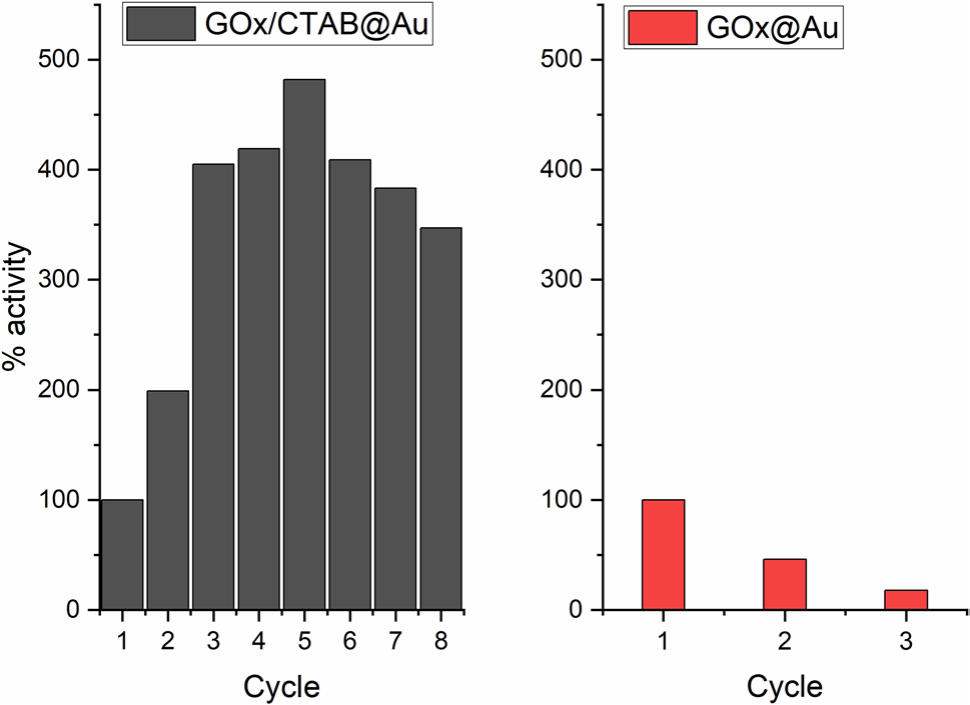
Recyclability of GOx/CTAB@Au (left) and of GOx@Au (right).

The co-entrapment of GOx with CTAB in gold also enables good recyclability—much better than the entrapment without the surfactant (Fig. 4), thus introducing a feature which is of relevance to various biotechnological applications. An interesting observation is that the activity increases significantly during the first cycles and remains much higher than the first cycles throughout the 8 tested cycles. The observation of increase in activity in the first few cycles is a known phenomenon in catalysts entrapped within porous matrices, such as doped sol–gel catalysts^32^, and has been ascribed to opening of blocked pores and widening of partially blocked pores from residual impurities, loosely held matrix nanoparticles.

## Discussion

**Figure 5:**
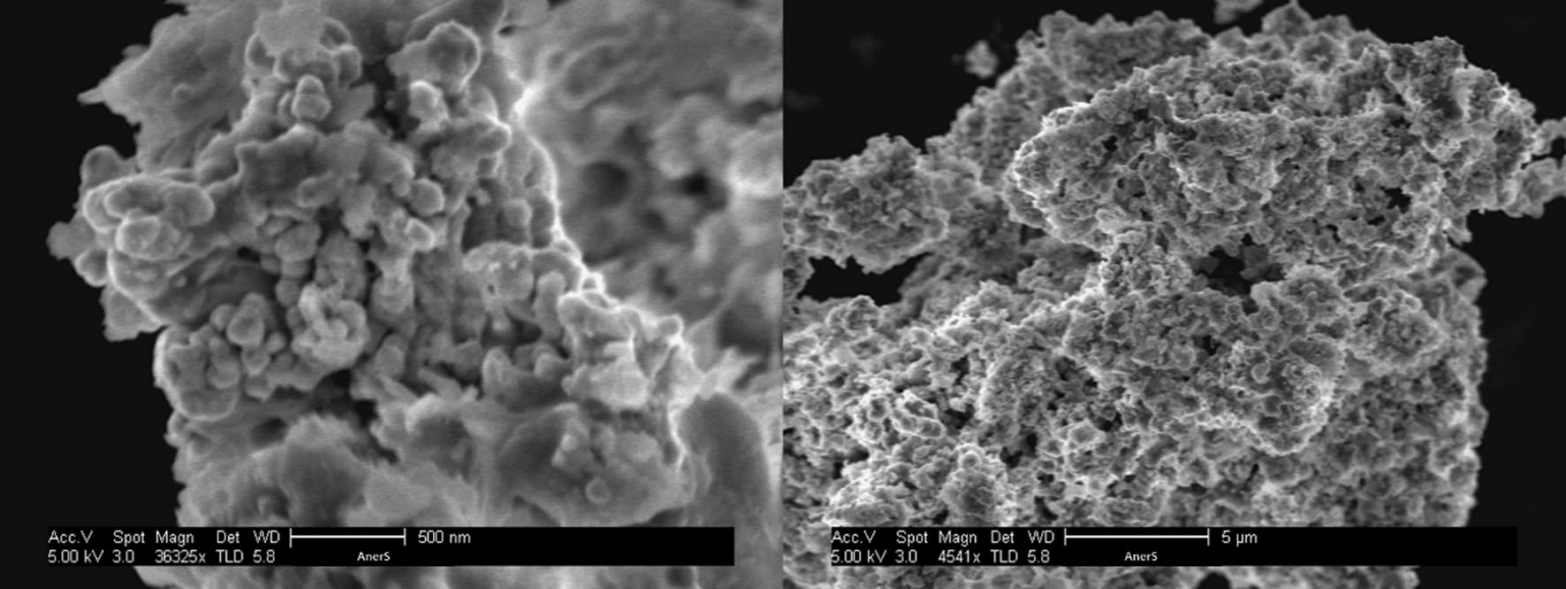
HR-SEM images of GOx/CTAB@Au (bars: left 500 nm, right: 5 µm).

**Figure 6:**
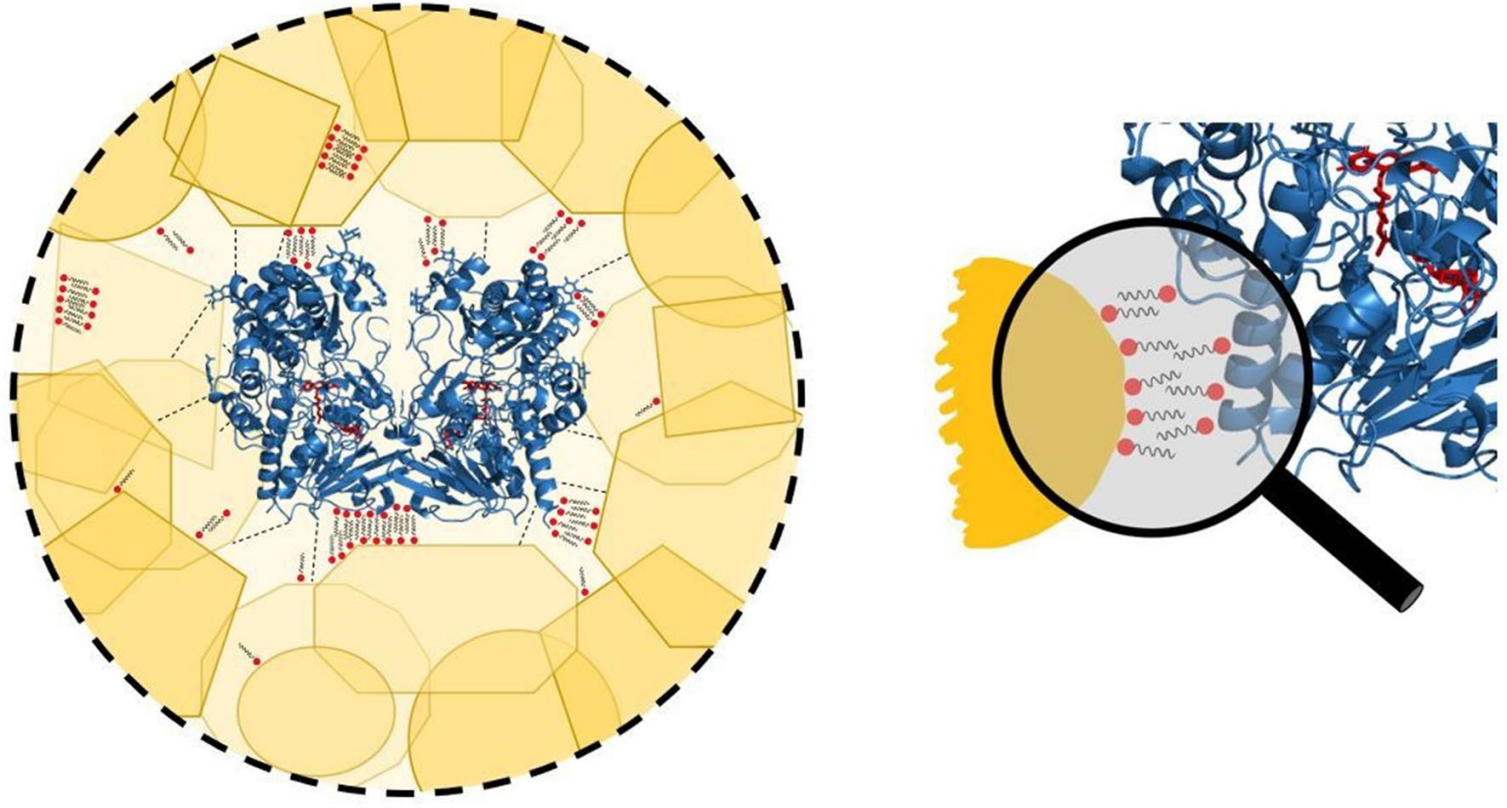
Left: illustration of the interactions which affect a suggested channel opening of the entrapped enzyme in gold with CTAB; right: zoom-in on the CTAB bilayer formed between the gold and GOx (RCSB ID: 1GPE).

What then enables the activity of the entrapped GOx at all? The gold matrix is porous with a hierarchical structure. The smallest building blocks are gold nanocrystals of around 16 nm (determined from XRD analysis^13^), which are tightly aggregated into sub-micron structures, which are further aggregated into micron-size particles, as can be seen in the HR-SEM imaging in Fig. 5. The surface area is ~ 30 m^2^/g, which is typical for porous materials, the porosity of which is interstitial. This porous structure allows diffusion of substrate molecules to the buried enzyme molecules, and diffusion of product molecules out, but the tight aggregation around the entrapped enzyme molecules prevents their leaching out (there is zero activity of the supernatant solutions). That property of efficient entrapment on one hand, and free molecular diffusion on the other hand is possible because the cage walls are perforated with interstitial pores which are too small for the enzyme to leach out, as illustrated in Fig. 6 (roughly, with an Au nanocrystallite average size of 16 nm, one gets interstitial pores to be around 4 nm, while GOx diameter is around 8 nm^33^). Adding to this physical entrapment property are the strong interactions of the enzyme with gold and CTAB, detailed below. The known cost of entrapment within a porous matrix is the diffusional limitation which slows the reaction rate, but then the gains are the enhanced stability (Fig. 3), the recyclability (Fig. 4), the ability to construct a device, and the prospect of modifying the activity.

In general, affecting and widening of enzyme activity has been achieved by two types of approach: tampering with the primary structure of the protein by various enzyme-engineering and mutation methods, which, from that point of view can be considered as forming new enzymes^34–36^; or, affecting the tertiary and quaternary conformational structure of the original native enzyme, leaving the sequence of amino acids untouched. Such conformational changes have been induced mainly by adsorptive interactions^37,38^. The main target of both approaches has been, as expected, the active site^36,38,39^, but far less attention has been devoted to affecting the channel leading to the active site. The rationale in approaching the (dynamic) channel is that it acts like a highly stereoselective separation column, allowing entrance to the active site only for a substrate that fits the stereoselective screening. Thus, changing the conformation of the channel—particularly widening that entrance—may lead to alteration of the selectivity; this, we propose below, is the main mechanism that explains the observations of this report. Affecting the channel was reported by enzyme engineering methods^40^, but affecting it by conformational changes that do not involve changes in the primary sequence, to the best of our knowledge, has not been reported.

The conformational changes that GOx undergoes in its entrapped state are due to three types of powerful interactions, each of which has been separately well documented, operating all together in the GOx/CTAB@Au system. The first is the interaction of proteins with gold. These interactions are with functional groups of most amino acids on the outer surface of the protein^41,42^, and particularly with the SH groups of the cysteine residues^43^, with the S of methionine side chain^44^, and with the exposed S–S moieties of cystine^45,46^ (Figs. S8, S9, Supplementary Material). Furthermore, since the buffered pH of the entrapped GOx—pH 5.1—is higher than its pI (4.2^47^) rendering the enzymes interface negatively charged, one should also consider interactions with the anionic aspartate and glutamate^41^.

The second interaction to consider is that of CTAB with gold. It is a well-known strong interaction^48,49^, which has been used, for instance, to control the morphology and shape of gold nano-structures^49–53^. A special feature of the CTAB-gold interaction, first proposed by El-Sayed et al.^49^ and confirmed by numerous subsequent studies, is the formation of an adsorbed CTAB double layer on the gold surface^49,54,55^. The proposed structure of that bilayer is the following (Fig. 6, right): in the first layer the cationic (Me_3_)R–N^+^ faces the gold surface through a bromide bridge^54^: Au–Br^**–**^–N^**+**^. Supporting that proposition is that fact that the bromide anion is known to adsorb strongly on gold (particularly on 111 planes but also on lower index plains^53^), and it has also strong attraction to CTA^+^^53^. The cetyl chain of CTAB, perpendicular to the gold surface, accepts the second layer through cetyl–cetyl hydrophobic interactions and a double-layer forms, much like in liposomes^55^: Au–Br^**–**^–N^**+**^–R–R–N^**+**^. That second layer terminal N^**+**^ of CTA^**+**^ is then free to interact with the negatively charged protein^56,57^, releasing the bromide. We assume that the bilayer structure is not perfect inside the gold cage, and that there are also isolated CTAB molecules adsorbed on the gold surface which do not take part in a bilayer—these may add hydrophobic interactions between the alkyl tail of the CTAB and the residues of the hydrophobic amino acids on the surface of the protein^58,59^, such as valine and leucine^59^. That holding of the enzyme inside the cage more rigidly by these combined types of interactions, is expressed by the major increase in the thermal stability (Fig. 3), and by the ability to recycle the entrapped enzyme (Fig. 4). But how do these interactions also affect the conversion of GOx into a general oxidase? This is discussed next:

The proposed induced conformational change is based on the fact that GOx is a homodimeric oligomer of which the two monomeric units are held by non-covalent bonds^60^. As common in many homodimeric enzymes, the interface between these two units forms the channel leading to the two active sites, a channel that evolved also to act as a stereoselective filter for substrates which can enter and reach the active site^61,62^. Unlike conformational changes which occur by 2D adsorption, which is a non-isotropic process, the 3D gold cage in our case, pulls apart the protein in an isotropic manner, that is, in all directions (Fig. 6). Since the interface between the two monomeric units is not held together by strong covalent bonds, it constitutes an initial “crack” that already exists in the enzyme, and which can be further affected. Thus, based on the activity analysis, we propose that a reasonable interpretation is that the combined pull of the direct protein-gold interaction and the gold-CTAB double layer interactions, opens that crack to a degree that the channel loses its evolutionary built strict stereoselective gate-keeper property, tailored for d-glucose exclusively. This, in turn, allows all substrates described in this report, to diffuse to the two active sites and reach them.

Further confirmation to this proposed mechanism comes from the comparison of the activity of GOx when entrapped with or without entrapment CTAB (a comparison possible for the monosaccharides which can be entrapped without CTAB). It is seen—Figs. 3, 4 and Table 2—that GOx/CTAB@Au greatly outperforms GOx@Au in any parameter, including even the performance on the native d-glucose. This comparison allows the two types of pulling effects—with pure gold, and through CTAB. In fact, that additional pull, overshadows the pull of the gold itself, as seen in Table 2. The observation that the partial widening of activity with gold only is generalized by co-entrapping CTAB, strengthens the proposed conformational opening the access to the active site. The focus on the channel is also highlighted by discussing what do these observations mean from the point of view of the active site—next.

**Figure 7:**
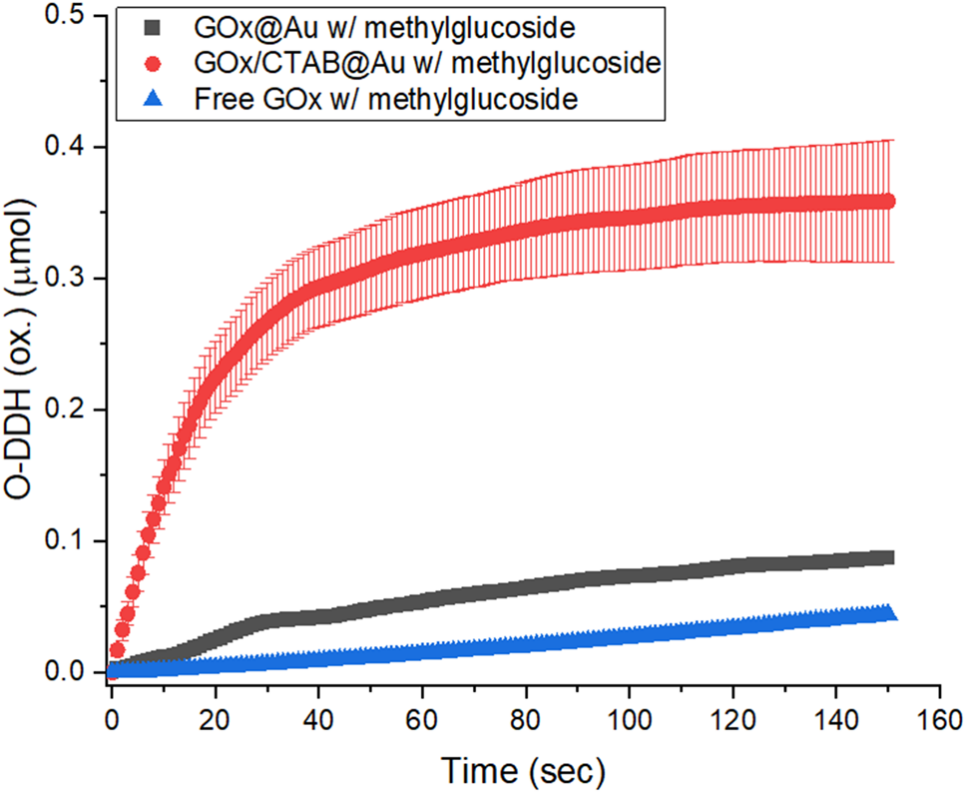
Activity of free GOx on methylglucoside: GOx@Au (red), GOx/CTAB@Au (black) and free GOx (blue) on methylglucoside (right).

Our observations indicate that once the access to the active site is opened to saccharides other than d-glucose, the active site is capable of oxidizing non-specifically other saccharides. That is, our observations indicate that selectivity of GOx is not a sole feature of the active site. Furthermore, these observations also suggest an interpretation that the active site of GOx (the peptide fragment containing Glu412, His516 and His559^63^) resembles an early-evolutionary preserved structure of this biocatalyst, and that the strict stereospecificity evolved over the ages with the build-up of the encompassing protein and its dimerization, to form the exclusive activity towards d-glucose of the modern enzyme we know. That non-specificity of the active site towards the stereochemistry of the saccharide CH–OH is particularly evident by the ability of the entrapped GOx to oxidize methyl glucoside and sucrose, two molecules in which the d-glucose analogous cyclic β-C(1)H-OH is blocked. That free GOx is capable of oxidizing methyl glucoside at all, utilizing air oxygen and releasing H_2_O_2_ is evident from the low but detectable activity in solution—Fig. 7. The efficient activity of the exposed active site is also compatible with the observation—Table 1—that the higher apparent K_M_ values observed of methyl glucoside, raffinose, sucrose and glucose-6-phosphate, are accompanied by higher V_max_ values: the lower affinity (higher K_M_) means shorter residence time at the active site, and that increases V_max_ if the oxidation step is fast.

In conclusion, “Au my GOx”: we have been able to convert GOx into a general sugar oxidase, including saccharides which lack natural specific oxidases. High-specificity and wide scope activity of enzymes are two desirable properties, which are complementary, and answer different needs in biotechnology, medicine, pharmaceutics, and enzymatic devices. Enzymes in their native form and environments usually answer the high-specificity requirement. And since this is the starting point, widening the scope of activity requires manipulation of the native enzyme. Here we have shown how adsorptive-induced conformational changes can be utilized for that purpose. As GOx is common and robust, and since most of the saccharides employed in this study are of high-volume industrial use but lack specific oxidases, our study opens new directions to be considered. For instance, one could envisage that GOx/air/d-glucose fuel cells^64^ can now be generalized to other common sugars or their mixtures, using GOx only^65^. An added advantage of the entrapment in gold developed in this study is that the protective heterogenization, renders the use of the general oxidative GOx easier to implement in the variety of potential uses.

## Methods

### Chemicals, enzymes and reagents

Sodium tetrachloroaurate(iii) dihydrate was purchased from Alfa Aesar. Zinc (granular, 20–30 mesh, ACS reagent, ≥ 99.8%) was purchased from Sigma Aldrich. Glucose oxidase (*A. niger*, ~ 135 U mg^−1^) and *O*-dianisidine (O-DDH) were purchased from Sigma Aldrich. d-Glucose was purchased from Honeywell Riedel-de Haën Research Chemicals. Glucose-6-phosphate, l-glucose, lactose, d-raffinose, methyl glucoside and sucrose were purchased from Sigma Aldrich. Fructose and xylose were purchased from Alfa Aesar. Peroxidase from horseradish (∼ 200 U mg^−1^) was purchased from Sigma Aldrich. Cetyltrimethylammonium bromide (CTAB) was purchased from Acros organics. Buffer (weight values are for a volume of 500 mL of buffer): 50 mM sodium acetate buffer (pH 5.1, 35 °C) was prepared for glucose oxidase by dissolving 2.05 g of sodium acetate anhydrous in distilled water, adjusting the pH to 5.1 with 1 M HCl solution.

### Entrapment of GOx in gold

161.5 mg of tetrachloroaurate(iii) dihydrate was dissolved in 3.49 mL of triple distilled water (TDW). Next, 4.10 mL of 50 mM sodium acetate buffer (pH 5.1) were added. Using 1 M NaOH, the pH was adjusted to neutral. Next, 1.0 mL of a mixture of 50 mM cetrimonium bromide (CTAB) and 0.1 mg/mL GOx was added to the reaction vial, followed by 40 mg of zinc granules. The reaction mixture was then stirred overnight at room temperature. The resulting GOx/CTAB@Au precipitate was filtered, washed and dried. The entrapment if full—this was assured by testing the activity of the supernatant solution and the washing solutions no activity was detected^13^. For comparative activity analysis purposes, entrapment without CTAB was carried out by following a similar procedure as follows: 161.5 mg of tetrachloroaurate(iii) dihydrate was dissolved in 3.49 mL of TDW, into which 4.10 mL of 50 mM sodium acetate buffer were also added. Next, the pH was adjusted to neutral pH by the addition on 1 M NaOH solution, followed by the addition of 1.0 mL of 0.1 mg/mL GOx solution. Finally, 40 mg of zinc granules were added, and the reaction mixture was stirred overnight at room temperature. The rest of the procedure was exactly as mentioned above^11^.

### Bioactivity assay and kinetics measurements

140 mg GOx@Au composite powder was placed in a polystyrene cuvette to which the following reagents were also added: 0.1 mL of horseradish peroxidase (0.3 mg mL^−1^, ~ 200 U mg^−1^), 0.1 mL of 50 mM sodium acetate buffer solution and a mixture of 2.4 mL 0.21 mM *O*-dianisidine solution (O-DDH) with 0.5 mL 10% (w/v) sugar solution*.* The enzymatic activity was measured spectrophotometrically by following the increase in absorbance at 500 nm, at 35 °C. For Michaelis–Menten analyses, the initial reaction velocities (V_0_) were determined from the initial slope of the kinetics plots and presented as normalized to the enzyme weight. “One enzymatic unit” is the amount of GOx which oxidizes 1 micromole of d-glucose to per minute.

### Control experiments

1. The activity of pure gold prepared by the same method without dopants, was checked and found to be zero^13^.
2. The entrapment of CTAB in gold, without an enzyme was tested for activity, to verify that the increase in activity was a result of the combination of all three components, and not due to some residual activity caused by the presence of CTAB on its own. This control experiment was conducted both with d-glucose and methylglucoside as substrates, and in both cases, it was seen that in order for a substantial activity to take place, the presence of the enzyme is pivotal.
3. The activity assay for the free enzyme in solution alongside CTAB—without gold—was tested. This was also performed on both d-glucose and methylglucoside as substrates, and in both cases, there was no increase in activity caused by the addition of CTAB, but rather a slight decrease in activity.

### Thermal stability and recyclability

For evaluation of the thermal stability, the composite powder was placed in a polystyrene cuvette that was temperature-equilibrated for 10 min to the desired temperature. Then were added 0.1 mL solution of 50 mM sodium acetate buffer, 0.1 mL of horseradish peroxidase (∼ 200 U mg^−1^, 0.3 mg mL^−1^, in TDW), 0.5 mL of 89.5 mM glucose solution, and 2.4 mL of 0.21 mM *O*-dianisidine solution, and the activity measured as previously described. The recyclability was evaluated by discarding the supernatant solution after each cycle, followed by the addition of all of the required components for the next cycle of activity.

### Instrumentation and protein data

Sirion (FEI) high-resolution SEM (scanning electron microscopy) was used for the analysis of high-resolution scanning electron microscopy. The measurements of UV–Vis (UV–visible) absorption were conducted using JASCO V-630 spectrophotometer. Protein structures for glucose oxidase were taken from the RCSB protein databank.

## Supplementary Information

Supplementary Information.

## Data Availability

The raw/processed data required to reproduce these findings cannot be shared at this time due to technical or time limitations, but can be provided upon request.
