# Supplementary material for: Glucose oxidase converted into a general sugar-oxidase
Source: Sci Rep. 2022 Jun 23;12:10716. doi: 10.1038/s41598-022-14957-6 (PMC9226012; doi:10.1038/s41598-022-14957-6)
Supplement: Supplementary file 1 — Supplementary Information. [file 41598_2022_14957_MOESM1_ESM.docx]

**Glucose oxidase converted into a general sugar-oxidase**

Yael Baruch-Shpigler and David Avnir*

Institute of Chemistry and the Center for Nanoscience and Nanotechnology, The Hebrew University

of Jerusalem, Jerusalem 9190401, Israel

**Supplementary Material**

1. Activity error bars:

The following plots are the activities of GOx/CTAB@Au on the various sugars, with error bars added.


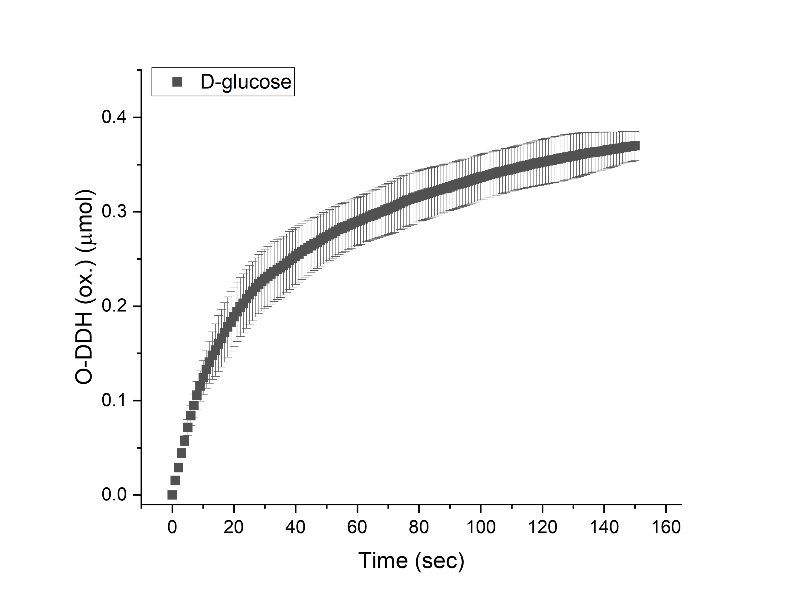


Fig. S1 – Activity of GOx/CTAB@Au on D-glucose


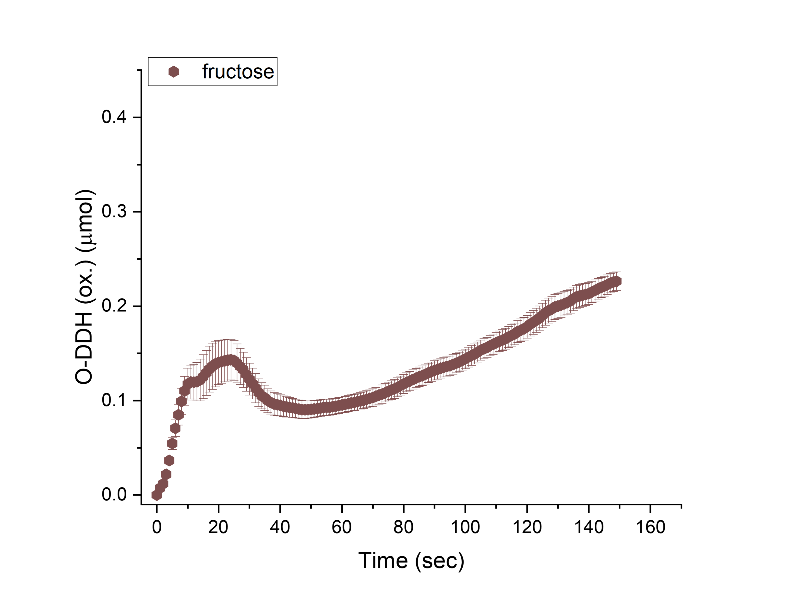


Fig. S2 – Activity of GOx/CTAB@Au on fructose


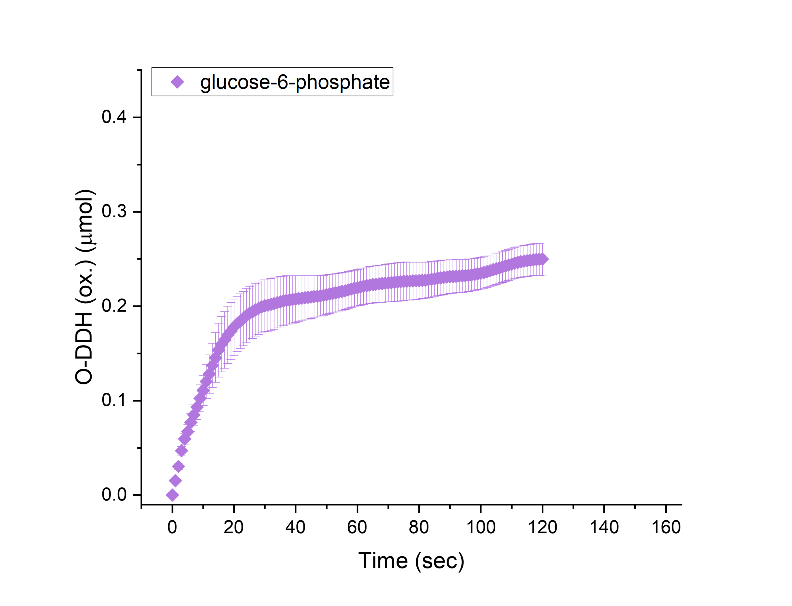


Fig. S3 – Activity of GOx/CTAB@Au on glucose-6-phosphate


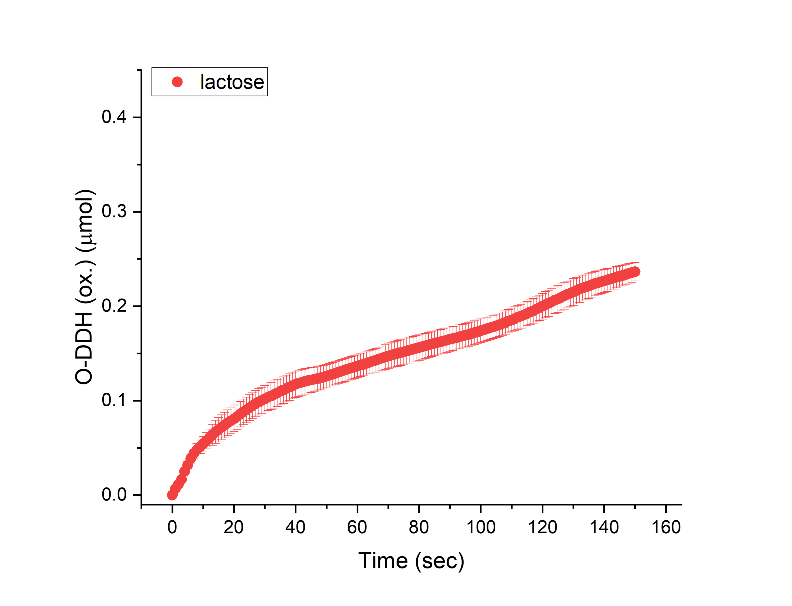


Fig. S4 – Activity of GOx/CTAB@Au on lactose


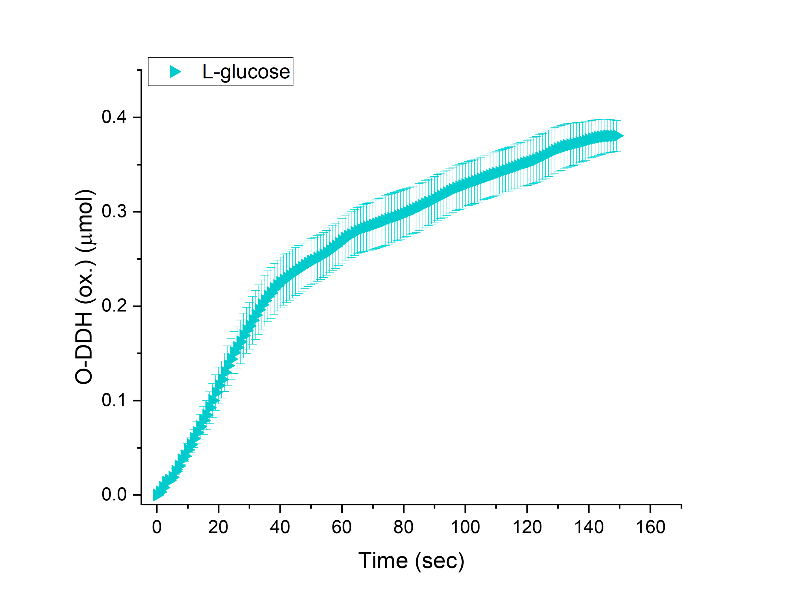


Fig. S5 – Activity of GOx/CTAB@Au on L-glucose


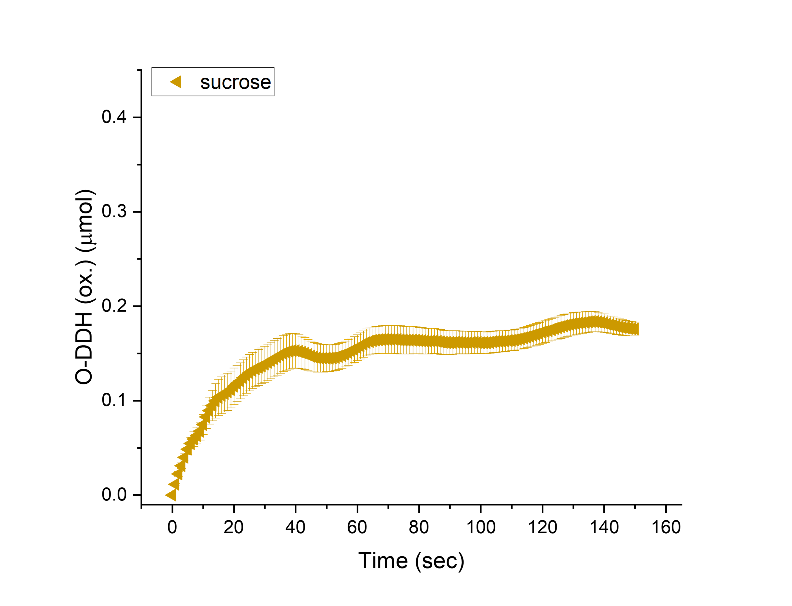


Fig. S6 – Activity of GOx/CTAB@Au on sucrose


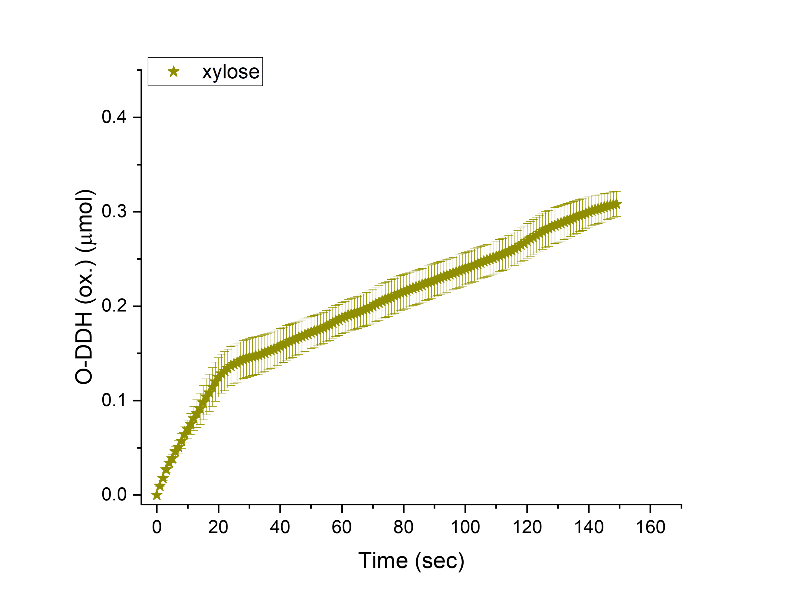


Fig. S7 – Activity of GOx/CTAB@Au on xylose

1. The interaction of S-containing amino acids with gold

In figure S8, the interaction between gold and cysteine is illustrated^1^. The reaction for the binding of the two entities is:

R-SH + Au 🡪 RS-Au + H_2_

These interactions are additional to the coordinative bonds between gold and thiol groups^2^, demonstrated in Fig. S9.


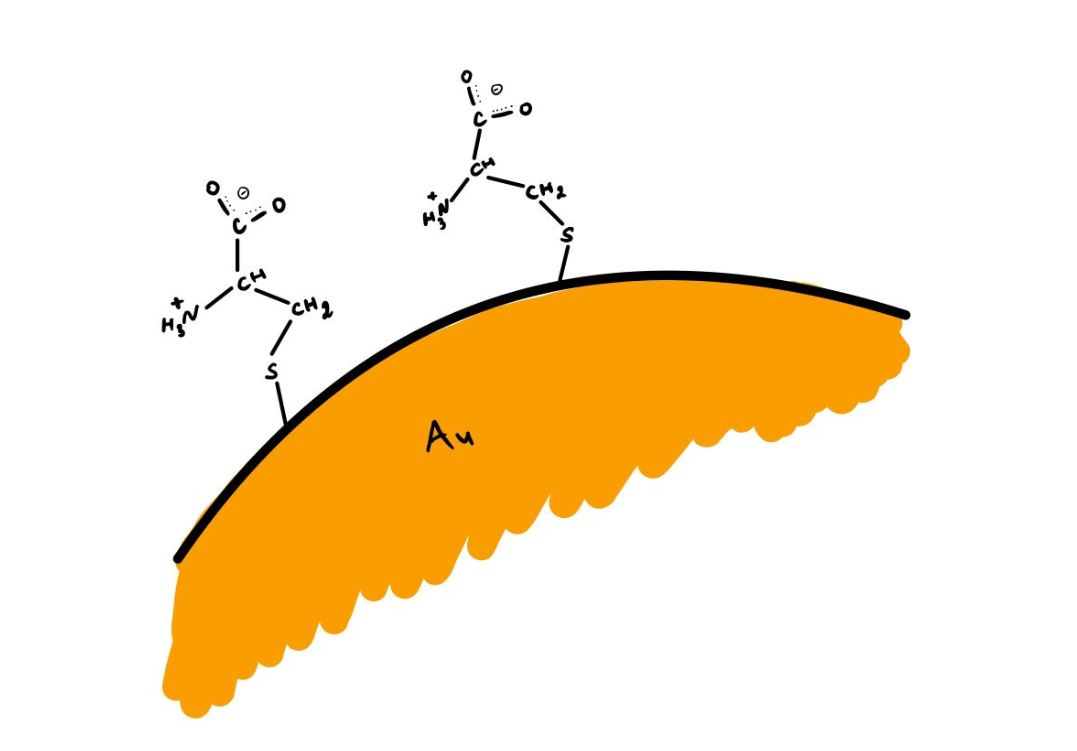


Fig. S8 – illustration of interaction between Au and sulfur group of cysteine amino acid


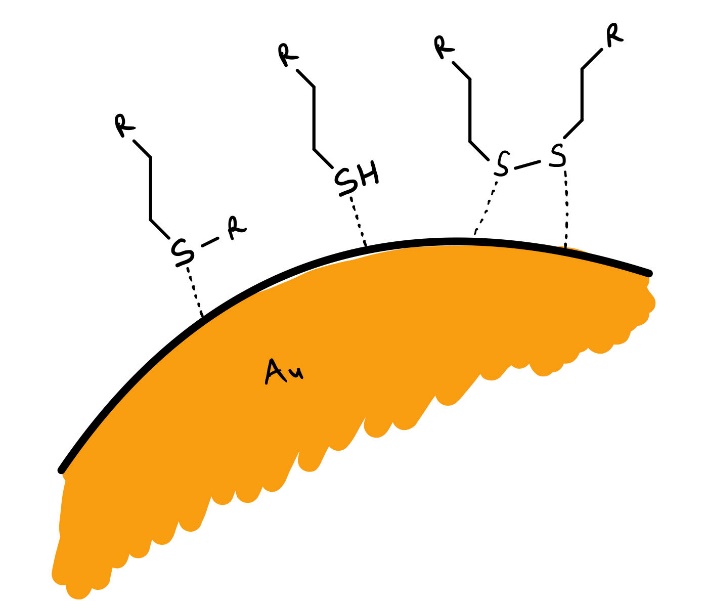


Fig. S9 – illustration of coordinative bonds between Au and sulfur groups of amino acids

Bibliography:

1. Abraham, A., Ilott, A. J., Miller, J. & Gullion, T. 1H MAS NMR study of cysteine-coated gold nanoparticles. *J. Phys. Chem. B* **116**, 7771–7775 (2012).

2. Inkpen, M. S. *et al.* Non-chemisorbed gold–sulfur binding prevails in self-assembled monolayers. *Nat. Chem. 2019 114* **11**, 351–358 (2019).
